# Assessing selectivity and bycatch of endangered, threatened and protected species in artisanal tuna fisheries in the NE Atlantic

**DOI:** 10.7717/peerj.21516

**Published:** 2026-10-30

**Authors:** Maite Louzao, Iñaki Oyarzabal, Iñigo Onandia, Jon Ruiz, Amaia Astarloa, Isabel García-Barón, Unai Cotano, Andrés Uriarte

**Affiliations:** AZTI, Marine Research, Basque Research and Technology Alliance (BRTA), Pasaia, Spain

**Keywords:** Fisheries bycatch, Migratory seabirds, Fishing selectivity, Artisanal tuna fisheries, Endangered, threatened and protected (ETP) species

## Abstract

Fisheries bycatch is a recognised pressure on marine megafauna, prompting international agreements to mandate monitoring and mitigation of impacts. The International Commission for the Conservation of Atlantic Tunas (ICCAT) specifically calls for systematic data collection on Endangered, Threatened and Protected species (ETPs), particularly in data-deficient regions. To address this need, the Spanish artisanal tuna fishery—comprising trolling and pole-and-line *métiers*—implemented an onboard observer programme in 2016 to document ETP bycatch in the Northeast Atlantic, an important migratory and overwintering area for marine megafauna. Our aims were to (1) assess fishery selectivity through catch and discard composition, (2) describe ETP species occurring in the fishing grounds and (3) characterise ETP bycatch events, including their frequency, the number of individuals involved per event, and the condition of animals upon release. The fishery showed high selectivity, with >99% of recorded catches consisting of target tuna species. Observers documented 18 ETP species occurring around fishing vessels (three marine mammals and 15 seabirds), illustrating substantial megafauna richness in the fishing grounds. ETP bycatch was limited to three seabird species: great shearwater *Ardenna gravis*, northern gannet *Morus bassanus* and northern fulmar *Fulmarus glacialis*. Great shearwaters accounted for most individuals recorded and were occasionally captured in events involving several birds during trolling operations, whereas northern gannets were recorded only in pole-and-line operations, where events consistently involved few individuals. A total of 52 individuals from three seabird species were recorded in 22 bycatch events, most of which were released alive. Spatial patterns reflected known species ecology, with shearwater bycatch concentrated in oceanic waters and gannet bycatch occurring primarily in shelf-slope areas. These findings provide the first systematic characterisation of ETP species occurrence and bycatch in this artisanal tuna fishery, supplying essential baseline information for ICCAT and supporting the development of monitoring approaches and management actions to reduce risks to ETP species in the Northeast Atlantic.

## Introduction

Fisheries bycatch is a major driver of marine megafauna decline (Lewison et al., 2014), with more than 20 million seabirds, turtles, marine mammals, sharks, rays and teleosts caught incidentally each year (Gray & Kennelly, 2018). Air-breathing species—including seabirds, marine mammals and sea turtles, many of which have little or no commercial value—are particularly vulnerable because they frequently become entangled or hooked in gear targeting more valuable species (Lewison et al., 2004). This vulnerability is further exacerbated in long-lived species with low reproductive rates and naturally high survival, for which even small increases in adult mortality can have severe population-level consequences (Finkelstein et al., 2010). As a result, many of these taxa are classified as Protected, Endangered or Threatened (ETP) under national and international conservation frameworks.

To address these concerns, multiple international conservation agreements and fisheries management regulations require member states to minimise impacts of fishing on non-target species, promoting an ecosystem approach to fisheries management. Examples include the FAO Code of Conduct, eco-labelling schemes, the EU Common Fisheries Policy and additional global, regional and national instruments (Gray & Kennelly, 2018). Within tuna and tuna-like fisheries, the International Commission for the Conservation of Atlantic Tunas (ICCAT) (2023) has explicitly called for systematic and standardised data collection on ETP bycatch for all fisheries under its mandate (ICCAT, bycatch programme, https://www.iccat.int/en/bycatch.html). However, assessing ETP bycatch remains challenging because many ETP species are highly mobile, migrating across vast oceanic regions and encountering multiple fleets and gear types along their routes (Lascelles et al., 2014). Their broad-scale movements increase exposure to different fisheries, thereby elevating the likelihood of bycatch and complicating attribution of impacts to specific fleets. A global assessment identified a major data gap in the Eastern Atlantic, where little information had been collected despite the presence of important industrial and small-scale fisheries (Lewison et al., 2014), echoing earlier findings (Tuck et al., 2011; Pott & Wiedenfeld, 2017). This supports ICCAT’s emphasis on improving observer coverage in the Atlantic, especially in productive waters where ETP species and fisheries co-occur (Kaschner & Pauly, 2005; Karpouzi, Watson & Pauly, 2007). Strengthening ETP bycatch assessment in these regions is therefore essential for advancing the ecosystem approach to fisheries management and for implementing effective monitoring programmes—such as dedicated observer schemes—that can systematically document ETP bycatch.

Within this broader context, it is essential to ensure systematic monitoring not only of industrial fleets but also of artisanal fisheries, so that bycatch across the full suite of ETP taxa can be properly documented and addressed. Evidence from small-scale fisheries indicates that marine mammals and sea turtles may occasionally be incidentally caught under certain environmental or operational conditions (Moore et al., 2010). These findings collectively underscore the need to incorporate artisanal tuna fisheries into formal bycatch-monitoring frameworks, ensuring that ETP bycatch—not only from industrial fleets—is adequately assessed in line with international conservation and reporting requirements.

In the Bay of Biscay (BoB) and adjacent waters of the Northeast Atlantic, an artisanal tuna fishery operates from ports along the northern Spanish coast (Santiago, 2004). This fishery, certified by the Marine Stewardship Council (MSC) since 2016, comprises two distinct *métiers*—trolling, which mainly targets albacore tuna (*Thunnus alalunga*), and pole-and-line, which targets both albacore and bluefin tuna (*T. thynnus*) (Santiago et al., 2016a). Although operationally different, these *métiers* are managed under the same certification unit and share common fishing grounds during the tuna season (June–September) (MSC, 2021). The albacore component of the fishery extends over a broad area (Sagarminaga & Arrizabalaga, 2010), whereas bluefin tuna fishing occurs predominantly in the southeastern BoB (Santiago et al., 2016b; Uranga et al., 2017). Occasional bycatch consists of skipjack (*Katsuwonus pelamis*) and bigeye tuna (*Thunnus obesus*) (de Zárate et al., 2005; de Zárate, Pérez & Quelle, 2015).

In this context, the present study aimed to generate *métier*-resolved evidence on fishery selectivity and ETP bycatch by: (1) assessing fishery selectivity through analyses of catch and discard composition, (2) describing the ETP species assemblage occurring around fishing operations (*i.e*., species present near vessels but not necessarily bycaught), and (3) characterising ETP bycatch events, including the species involved, the frequency of events, their intensity (number of individuals per event), and the condition and immediate fate of individuals upon release (alive, injured or dead). Together, these objectives provide a comprehensive understanding of how protected species occur and are bycaught in the fishery, strengthen ETP bycatch assessment at the *métier* scale, and help address the evidence gap identified for the Northeast Atlantic in line with ICCAT’s monitoring and reporting guidance. Research on ETP bycatch in this fishery remains at an early stage, and detailed fishery- and region-specific information is still scarce. To date, only recent risk-focused analyses (García-Barón et al., 2023) offer preliminary insights for informing practical conservation and management measures. Generating detailed, context-specific information such as that provided here is essential for developing effective, proportionate and operationally realistic mitigation strategies that support both ETP conservation objectives and the long-term sustainability of artisanal tuna fisheries.

## Materials and Methods

### Study fleet

The Spanish artisanal tuna fishery operating in the Bay of Biscay (BoB) and adjacent oceanic waters of the Northeast Atlantic comprises numerous small vessels based along the northern Spanish coastline, from the Basque Country to Galicia. The fleet encompasses two main *métiers*—trolling and pole-and-line—whose operational characteristics are summarised in Fig. 1 and Table 1. Fleet size varies annually, averaging approximately 450 vessels, with ~90% employing trolling gear and ~10% using pole-and-line (de Zárate & Parejo, 2021).

**Figure 1:**
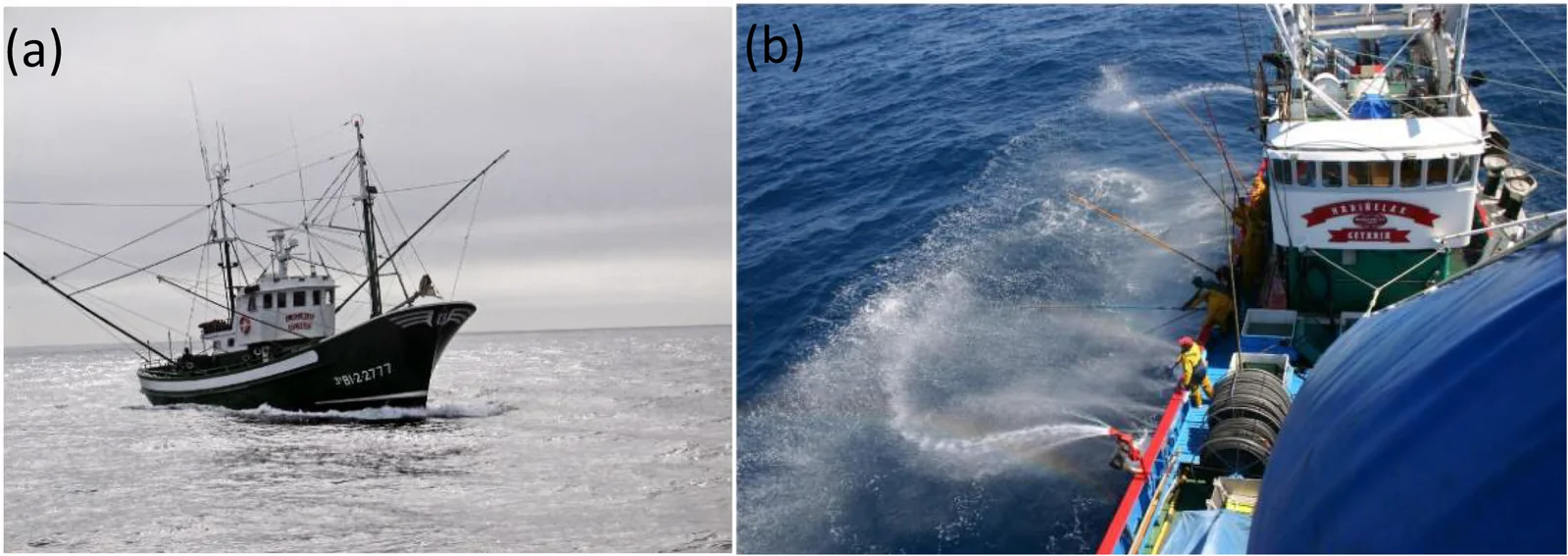
Illustrations describing the fishing operations of the artisanal tuna fishing in the Bay of Biscay. (A) The trolling *métier*; (B) the pole-and-line *métier*. Photograph sources: Iñigo Onandia.

**Table 1:** Summary of operational characteristics of the trolling and pole-and-line *métiers* in the Bay of Biscay artisanal tuna fishery. ICCAT SCRS Manual descriptions were used as general guidance; Bay of Biscay-specific details obtained from AZTI expert knowledge (AZTI, 2024, personal communications).

Parameter	Trolling	Pole-and-line
Target species	Mainly albacore	Albacore and bluefin
Number of lines/rods	Up to 15 lines (hydraulic reels)	6–7 rods (5–6 m)
Hook type and size	Double hooks, size 3/0	Single hooks, size 3/0–4/0
Number of hooks	One double hook per line	One hook per rod
Bait/lure type	Artificial lures only (plastic squid-type)	Live small pelagic fish (anchovy, sardine, horse mackerel, small mackerel)
Mean operational depth	Surface layer (planing at surface)	Surface layer (near vessel)
Fishing speed/mode	~7 knots, continuous towing	Stationary/slow drift during chumming
Length of deployed line	Long towed lines behind vessel (variable)	Short lines held directly beside vessel
Typical fishing grounds	Oceanic & shelf-slope waters (>200 m)	Shelf-slope and coastal-proximal tuna aggregation zones

The trolling *métier* typically operates with up to 15 fishing lines deployed from hydraulic reels (Fig. 1). Each line is equipped with an artificial lure—generally a plastic squid-type imitation—rigged with a double 3/0 hook. Lures are towed at approximately 7 knots, which keeps them planning at the sea surface; thus, the gear operates within the uppermost centimeters of the water column (AZTI, 2024, personal communications). The primary target species is albacore tuna, although bigeye and bluefin tuna may occasionally be caught. Because this *métier* does not hold a bluefin quota, any bluefin tuna bycaught must be released alive, unless brought on board dead.

The pole-and-line *métier*, in contrast, relies on the use of live bait to attract tuna schools to the vessel. Vessels generally deploy 6–7 rods, each 5–6 m in length and fitted with a single 3/0–4/0 hook selected according to the size class of tuna being targeted. These hooks operate at the surface while schooling tuna are enticed towards the vessel by live bait. Bait acquisition occurs prior to tuna fishing, using small purse-seine sets designed to capture limited quantities of baitfish while maintaining their physical condition. Common bait species include anchovy (*Engraulis encrasicolus*), sardine (*Sardina pilchardus*), juvenile Atlantic horse mackerel (*Trachurus trachurus*) and Atlantic mackerel (*Scomber scombrus*). Once caught, baitfish are transferred alive to onboard live wells until use. During baiting operations, slipping (discarding fish from the purse seine) is performed when necessary, and most individuals are released alive. Previous studies have reported high post-release survival due to the small set size and low stress associated with bait purse-seine maneuvers (Arregi et al., 2014; Scientific, Technical and Economic Committee for Fisheries (STECF), 2014). Pole-and-line vessels based mainly in Basque Country ports primarily target albacore and bluefin tuna, with occasional captures of bigeye tuna. Albacore landings mostly comprise migrating juveniles (1–3 years old) (Chust et al., 2019; de Zárate & Parejo, 2021), whereas bluefin tuna catches are dominated by juveniles with the presence of some adults ≥4 years old (Rodríguez-Marín et al., 2003; Cort & Abaunza, 2019).

### Data collection

The onboard observer programme operated from 2016 to 2019, although the temporal coverage differed between *métiers*. Trolling activity was monitored from 2017 to 2019 in vessels from the Basque Country and Asturias, while pole-and-line operations were monitored throughout 2016–2019 in the Basque Country fleet. Importantly, the at-sea monitoring programme was specifically designed to document both fishery selectivity and ETP bycatch, rather than functioning as a generalised observer scheme. This focused design ensured consistent and systematic recording of vessel operations, gear characteristics and the spatial distribution of fishing effort, generating robust *métier*-specific datasets for catch, discard and bycatch analyses.

Vessel selection was based on fisher collaboration and the availability of adequate working space for embarked observers. Observers monitored 100% of fishing operations, systematically recording tuna and non-tuna catches, all ETP bycatch events and the condition of each bycaught individual. A complete list of monitored fishing trips is provided in Table S1. Additionally, observers conducted opportunistic sightings of ETP species immediately before and after fishing operations, as well as during selected transits, using an elevated vantage point to maximise the field of view. Meteorological conditions—such as wind strength and sea state—were recorded concurrently to contextualise sighting and bycatch events.

For this study, ETP species were defined according to the functional groups prioritised under Biodiversity Descriptor 1 of the Marine Strategy Framework Directive (MSFD; 2008/56/EC), comprising seabirds, marine mammals and sea turtles. ETP occurrence and bycatch were analysed following a two-step framework. First, ETP occurrence in the fishing grounds was characterised using the opportunistic sighting data. Second, ETP bycatch was evaluated based on recorded capture events, each of which was defined as an ETP capture occurring at a unique combination of latitude, longitude and time. A single event could therefore involve one or several individuals, and in some cases more than one ETP species. Event intensity was defined as the number of individuals captured within a single event. The spatial distribution of fishing effort and ETP bycatch was subsequently mapped at the scale of individual fishing operations.

### Data analysis

Fishery selectivity was assessed by summarising catch and discard composition for each *métier*, expressed as the proportion of retained and non-retained individuals by species. ETP occurrence was summarised using opportunistic sighting data recorded by observers, and used to describe the assemblage of species present in the fishing grounds. ETP bycatch was analysed descriptively by summarising capture events. Event rate was calculated as the proportion of monitored fishing days with at least one bycatch event for each *métier*, and event size as the number of individuals per event. Species-specific summaries included the number of events and total individuals recorded for each species. Event totals do not sum across species because events may involve multiple individuals or species.

Spatial representation was conducted using GPS positions recorded for each fishing operation, and maps of fishing effort, and ETP bycatch were produced using QGIS (version 3.34.10). Descriptive summaries and visualisations—including histograms of the number of individuals per event, species-level contributions to bycatch and release-condition summaries—were generated in R (version 4.4.1). Given the very small number of events and individuals, and the highly uneven distribution of bycatch across species and *métiers*, no inferential statistical analyses were conducted.

### Conservation status of endangered, threatened and protected species

The conservation status of all ETP species recorded in this study was determined through a suite of internationally recognised legislative frameworks and conservation lists. At the national level, species status was derived from the List of Wild Species under a Special Protection Regime and the Spanish Catalogue of Threatened Species (RD 139/2011) (Blanco & González, 1992). At the European level, seabird conservation status followed the EU Birds Directive (2009/147/EC), while marine mammals and sea turtles were assessed under the EU Habitats Directive (92/43/EEC). ETP species were further evaluated within the context of Biodiversity Descriptor 1 of the MSFD, which prioritises these taxa for monitoring, and the OSPAR List of Threatened and/or Declining Species and Habitats, identifying regional conservation priorities for the Northeast Atlantic. Finally, global conservation status was obtained from the IUCN Red List of Threatened Species, providing internationally standardised assessments of extinction risk.

## Results

### Fishery selectivity

A total of 76 fishing trips from the artisanal tuna fishery were monitored, corresponding to 677 sampling days (Fig. 2). Fifteen trolling trips—13 from the Basque Country and two from Asturias—were observed, covering 243 fishing days between 2017 and 2019. This represents ~1.7% of the total estimated fishing days for this *métier* (Table 2). Across monitored trips, 226 tonnes of albacore tuna were landed (Table 2), and catches were highly selective, with 99.86% of the retained catch consisting of albacore. Discards were minimal, limited to non-retained tuna species such as Atlantic bluefin tuna (0.03%) and bigeye tuna (0.08%). Additional isolated captures included a white marlin (*Tetrapturus albidus*) in 2018 and an Atlantic bonito (*Sarda sarda*) in 2019. All non-retained individuals were either released alive or kept for crew consumption due to the absence of quota for these species.

**Figure 2:**
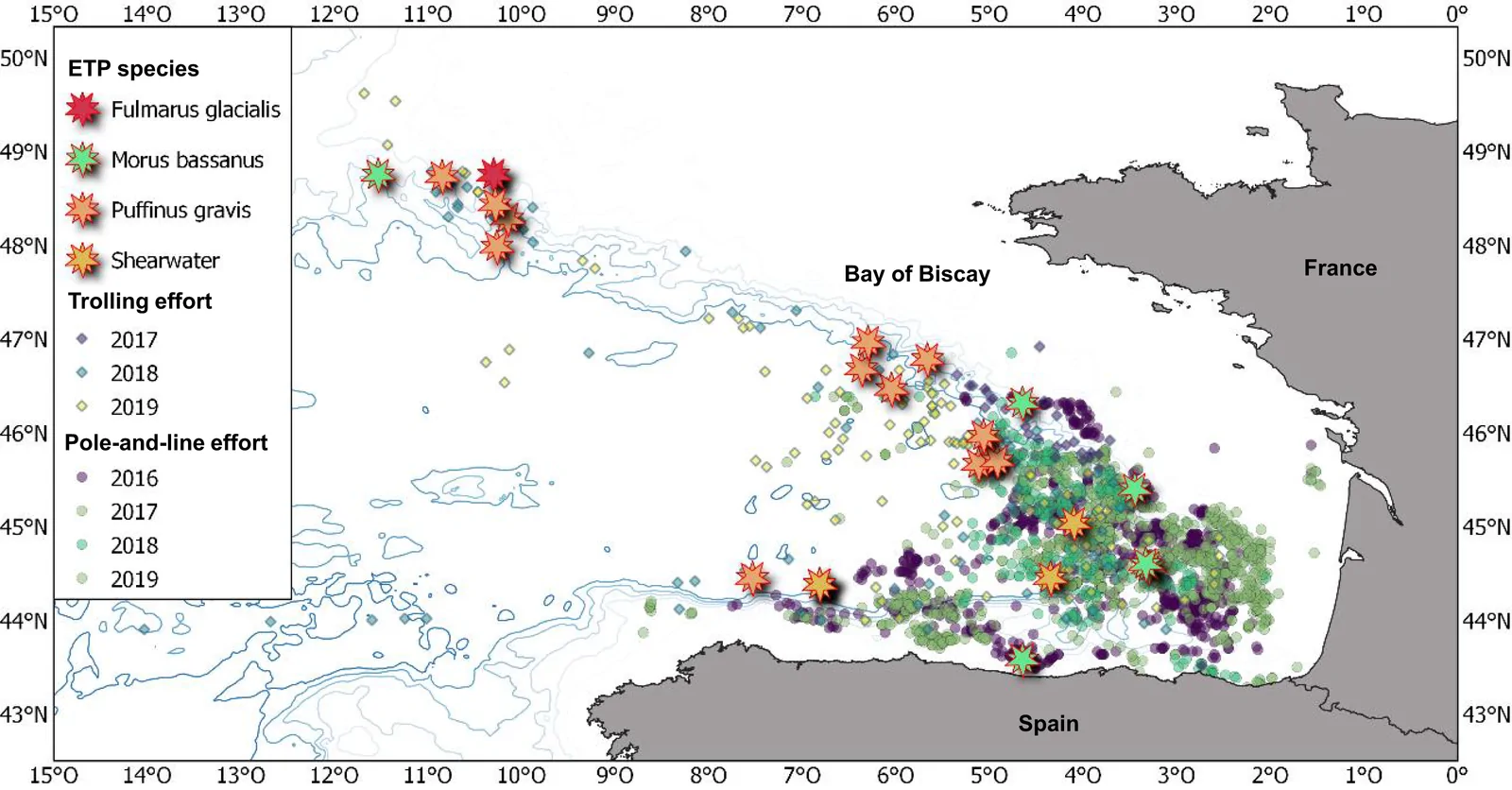
Map showing the monitoring of the artisanal tuna fishery in the Bay of Biscay during the 2016–2019 period. Spatial location of the trolling and pole-and line fishing (points and squares, respectively) by year. The bycatch events of endangered, threatened and protected (ETP) species are as well represented (Northern fulmar *Fulmarus glacialis*, Northern gannet *Morus bassanus*, Great shearwater *Ardenna gravis*, unidentified shearwaters).

**Table 2:** Summary of trolling fishing selectivity and bycatch of Endangered, Threatened and Protected (ETP) species.

Year	N of monitored fishing trips	N monitored days (% of total fleet fishing days)	Total catch	Discards				ETP bycatch									
			ALB	BET	BFT	BON	WHM	Northern gannet		Great shearwater		Shearwater Spp.		Northern fulmar		Total ETP	
			Kg (N ind)	Kg (N ind)	Kg (N ind)	Kg (N ind)	Kg (N ind)	N event	N Ind	N event	N Ind	N event	N Ind	N event	N Ind	N event	N Ind
2017	2	32 (0.8%)	24,595 (4,688)	0	0	0	0	0	0	0	0	0	0	0	0	0	0
2018	7	113 (1.6%)	112,356 (18,397)	163 (13)	22 (5)	0	20 (1)	2	2	11	35	0	0	1	1	14	38
2019	6	98 (2.8%)	89,235 (11,690)	48 (3)	72 (16)	3 (1)	0	3	3	0	0	3	8	0	0	6	11
Total	15	243 (1.7%)	226,186 (34,775)	211 (16)	94 (21)	3 (1)	20 (1)	5	5	11	35	3	8	1	1	15*	49

**Note:**

Trolling tuna fishing trips (including the percentage of coverage relative to the total fishing days of the fleet), landed catches and discards recorded during the 2016–2019 monitoring period, as well as the number of individuals of ETP species incidentally caught. Fish species are identified by their FAO codes: ALB, Albacore tuna (*Thunnus alalunga*); BET, Bigeye tuna (*Thunnus obesus*); BFT, Atlantic bluefin tuna (*Thunnus thynnus*); BON, Atlantic bonito (*Sarda sarda*); WHM, Atlantic white marlin (*Tetrapturus albidus*). *N*, number; *Ind*, individuals. *The total number of ETP-bycatch events represents unique combinations of latitude, longitude, and time. Because a single event may involve more than one species, the total number of events does not equal the sum of species-specific events.

A total of 61 pole-and-line trips were monitored between 2016 and 2019, covering 434 fishing days, accounting for ~3.3% of the total fishing days for this *métier* (Table 3). Monitored trips landed more than 1,040 tonnes of tuna, consisting mainly of albacore (93.7%), followed by bluefin tuna (5.4%) and bigeye tuna (0.7%). No discards occurred during tuna fishing, because all retained individuals belonged to quota-regulated species. Minor discards occurred only during live-bait purse-seine operations. A total of 26,669 kg of baitfish were caught alive, primarily anchovy (77.2%), followed by Atlantic horse mackerel (5.7%), Atlantic mackerel (1.7%), and sardine (2.6%). A fraction of this bait was discarded—mostly alive through slipping—which represented 14.9% of the bait catch or 0.36% of the total catch from monitored trips. Species most frequently released alive included: anchovy, sandy swimming crab (*Liocarcinus depurator*), common stingray (*Dasyatis pastinaca*), garfish (*Belone belone*), greater weever (*Trachinus draco*), blue shark (*Prionace glauca*), and sunfish (*Mola mola*).

**Table 3:** Summary of pole-and-line selectivity and bycatch of Endangered, Threatened and Protected (ETP) species.

Year	N of monitored fishing trips	N monito-red days (% of total fleet fishing days)	Total catch			Bycaught ETPs									
			ALB	BFT	BET	Northern gannet		Great shearwater		Shearwater Spp.		Northern fulmar		Total ETP	
			Kg (N ind)	Kg (N ind)	Kg (N ind)	N event	N Ind	N event	N Ind	N event	N Ind	N event	N Ind	N event	N Ind
2016	27	180 (4.2%)	315,682 (54,232)	39,489 (679)	132 (14)	2	4	0	0	0	0	0	0	2	4
2017	20	151 (3.4%)	285,801 (57,304)	12,587 (130)	7,483 (280)	0	0	0	0	2	2	0	0	2	2
2018	8	60 (2.6%)	211,178 (27,545)	4,195 (67)	0	0	0	3	4	0	0	0	0	3	4
2019	6	43 (2.0%)	165,503 (11,186)	787 (6)	20 (2)	0	0	0	0	0	0	0	0	0	0
ALL	61	434 (3.3%)	978,163 (173,859)	57,058 (882)	7,635 (296)	2	4	3	4	2	2	0	0	7	10

**Note:**

Pole-and-line tuna fishing trips (including the percentage of coverage relative to the total fishing days of the fleet), landed catches and discards recorded during the 2016–2019 monitoring period, as well as the number of ETP species incidentally caught. Fish species are identified using their FAO codes: ALB, Albacore tuna (*Thunnus alalunga*); BET, Bigeye tuna (*Thunnus obesus*); BFT, Atlantic bluefin tuna (*Thunnus thynnus*). *N*, number; *Ind*, individuals.

### ETP species occurrence and bycatch

Observers recorded a total of 18 ETP species associated with fishing operations (Table 4), comprising three marine mammals—the fin whale (*Balaenoptera physalus*), the common dolphin (*Delphinus delphis*), and the killer whale (*Orcinus orca*)—as well as fifteen seabird species, such as shearwaters (great shearwater *Ardenna gravis*, sooty shearwater *A. grisea*, Cory’s shearwater *Calonectris borealis*, Balearic shearwater *Puffinus mauretanicus*, Manx shearwater *P. puffinus*, northern fulmar *Fulmarus glacialis*), storm-petrels (European storm-petrel *Hydrobates pelagicus*, Wilson’s storm-petrel *Oceanites oceanicus*, band-rumped storm-petrel *H. castro*), gulls (yellow legged-gull *Larus michahellis*, lesser black-backed gull *L. fuscus*, Sabine’s gull *Xema sabini*), terns (common tern *Sterna hirundo*, Sandwich tern *Thalasseus sandvicensis)* and northern gannets *Morus bassanus*.

**Table 4:** Summary of endangered, threatened and protected (ETP) species associated with, and incidentally bycaught in, the artisanal tuna fishery.

Taxonomic group	Group	Common name	Scientific name	Bycaught
Mammal		Fin whale	*Balaenoptera physalus*	
Mammal		Common dolphin	*Delphinus delphis*	
Mammal		Killer whale	*Orcinus orca*	
Bird	Shearwater	Great shearwater	*Ardenna gravis*	*+*
Bird	Shearwater	Sooty shearwater	*Ardenna grisea*	
Bird	Shearwater	Cory’s shearwater	*Calonectris borealis*	
Bird	Shearwater	Balearic shearwater	*Puffinus mauretanicus*	
Bird	Shearwater	Manx shearwater	*Puffinus puffinus*	
Bird	Shearwater	Northern fulmar	*Fulmarus glacialis*	*+*
Bird	Storm-petrel	European storm-petrel	*Hydrobates pelagicus*	
Bird	Storm-petrel	Wilson’s storm-petrel	*Oceanites oceanicus*	
Bird	Storm-petrel	Band-rumped storm-petrel	*Hydrobates castro*	
Bird	Gull	Yellow legged-gull	*Larus michahellis*	
Bird	Gull	Lesser black-backed gull	*Larus fuscus*	
Bird	Gull	Sabine’s gull	*Xema sabini*	
Bird	Tern	Common tern	*Sterna hirundo*	
Bird	Tern	Sandwich tern	*Thalasseus sandvicensis*	
Bird	Gannet	Northern gannet	*Morus bassanus*	*+*

**Note:**

Endangered, threatened and protected (ETP) species observed within the fishing grounds of artisanal tuna vessels in the Bay of Biscay during the 2016–2019 monitoring period. Species incidentally caught in fishing gears are indicated with a “+” in the last column.

ETP bycatch exhibited a highly sparse and irregular pattern across *métiers* (Fig. 3). Most monitored fishing days showed no captures, and when bycatch occurred, events generally involved a single individual, although several multi-individual events were also recorded. Across 243 monitored trolling days, observers documented 15 distinct bycatch events, involving a total of 49 individuals (Table 2). Across 434 monitored pole-and-line days, seven events were recorded, involving 10 individuals (Table 3). These values correspond to bycatch occurrence on 6.2% of monitored trolling days and 1.6% of monitored pole-and-line days, confirming a higher frequency of events in trolling under the monitoring effort achieved. Importantly, the total number of events reflects unique combinations of latitude, longitude, and time; because individual events may involve more than one species, the overall number of events does not represent the sum of species-specific events.

**Figure 3:**
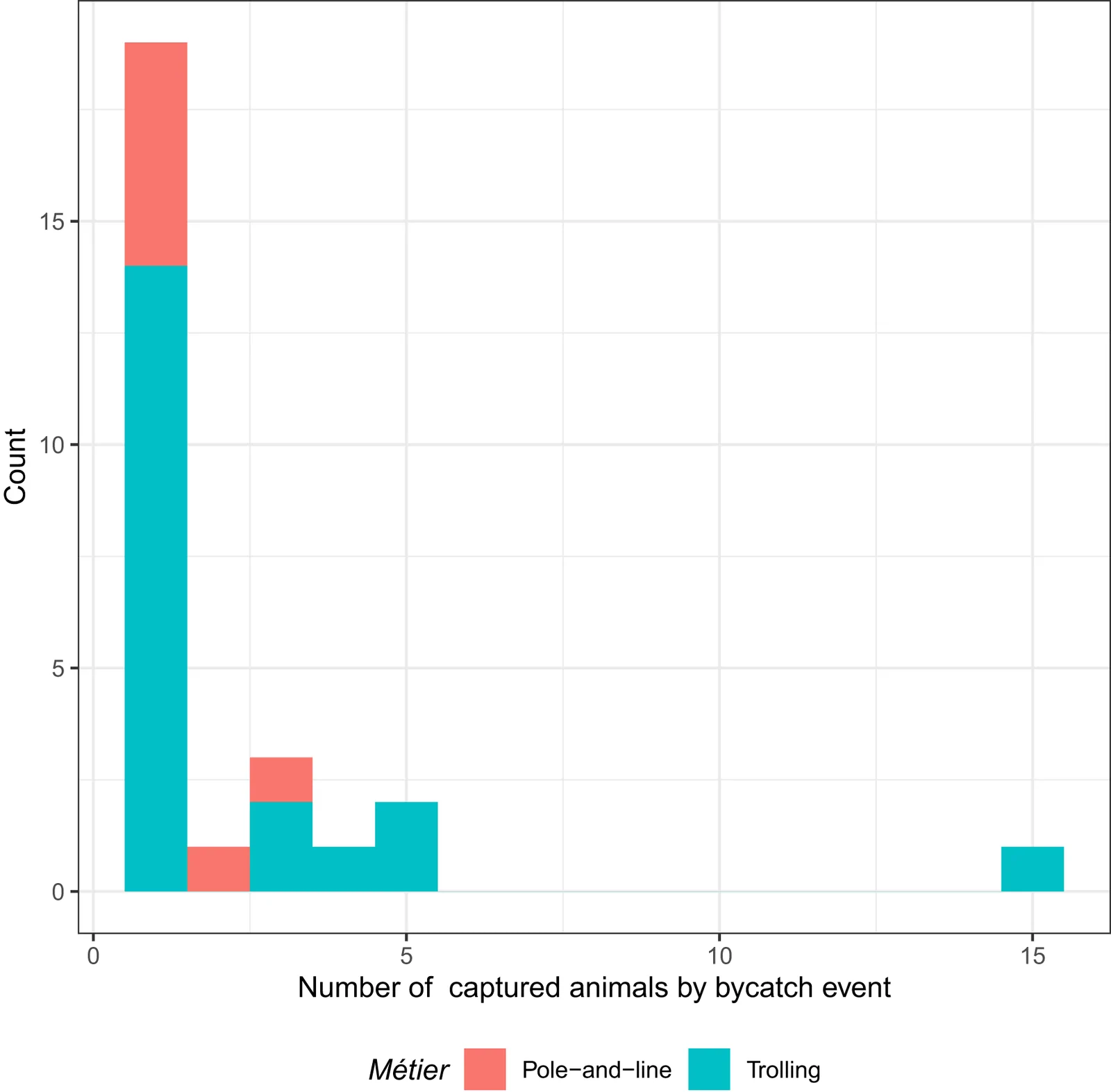
Bycatch of endangered, threatened and protected animals in the artisanal tuna fishery of the Bay of Biscay (2016–2019). The histogram shows the number of captured animals by bycatch event by *métier*.

Although 18 ETP species were observed around vessels, bycatch was limited to three seabird species—the great shearwater, the northern gannet, and the northern fulmar—plus a group of unidentified shearwaters (Tables 2 and 3). The composition of ETP bycatch is shown in Fig. 4. Great shearwaters represented the largest share of bycatch, with a total of 14 events involving 39 individuals across the fishery, including 11 events (35 individuals) recorded in the trolling *métier* in 2018 and three events (four individuals) in pole-and-line in 2018. Northern gannets were recorded in both *métiers*, with two events (two individuals) in trolling in 2018 and three events (three individuals) in 2019, and two events (four individuals) in pole-and-line in 2016, totalling seven events and nine individuals. Unidentified shearwaters accounted for three events (eight individuals) in trolling (2019) and two events (two individuals) in pole-and-line (2017), totalling five events and ten individuals. A single northern fulmar was recorded in one event during trolling in 2018. Given the small sample sizes—often fewer than ten individuals—these proportions should be interpreted with caution. Although marine mammals were recorded during vessel observations, bycatch was limited to seabirds, with no capture events documented for this taxa.

**Figure 4:**
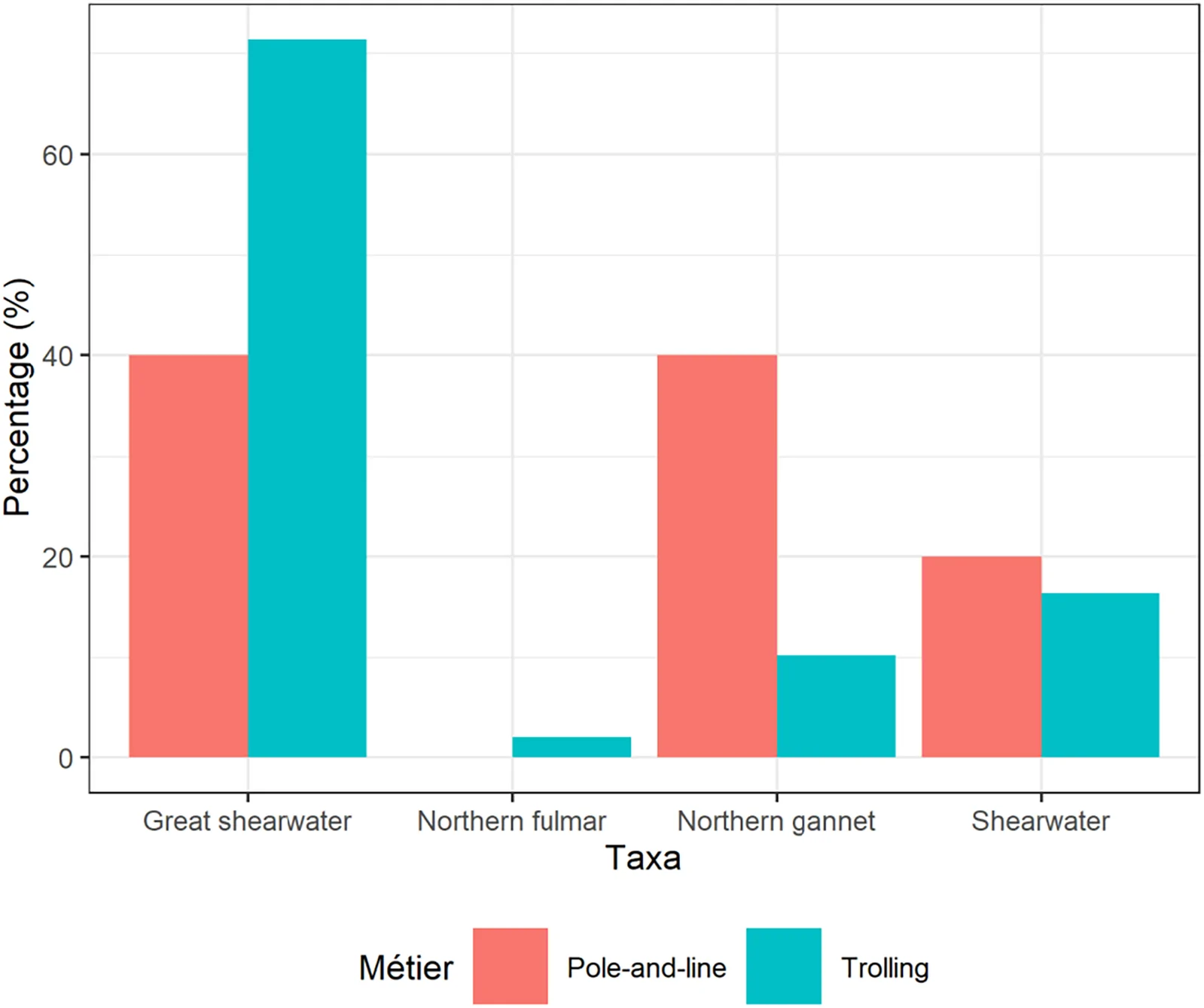
Identification of bycaught taxa in the artisanal tuna fishery of the Bay of Biscay (2016–2019). Bycatch is characterised by the percentage of individuals incidentally captured during the monitoring programme.

Shearwater captures occurred predominantly from late July to September, mainly in oceanic waters >200 m depth (Fig. 2), whereas northern gannet captures occurred in shelf-slope waters during summer pole-and-line operations (June–September). Bycaught birds were typically hooked externally—most often on the wing or beak—and were released alive (Fig. 5). Release rates were 92.3% for great shearwaters, 100% for unidentified shearwaters, 57% for northern gannets, and 100% for the single northern fulmar recorded. Given the small sample sizes for several taxa—often fewer than ten individuals—these proportions should be interpreted with caution.

**Figure 5:**
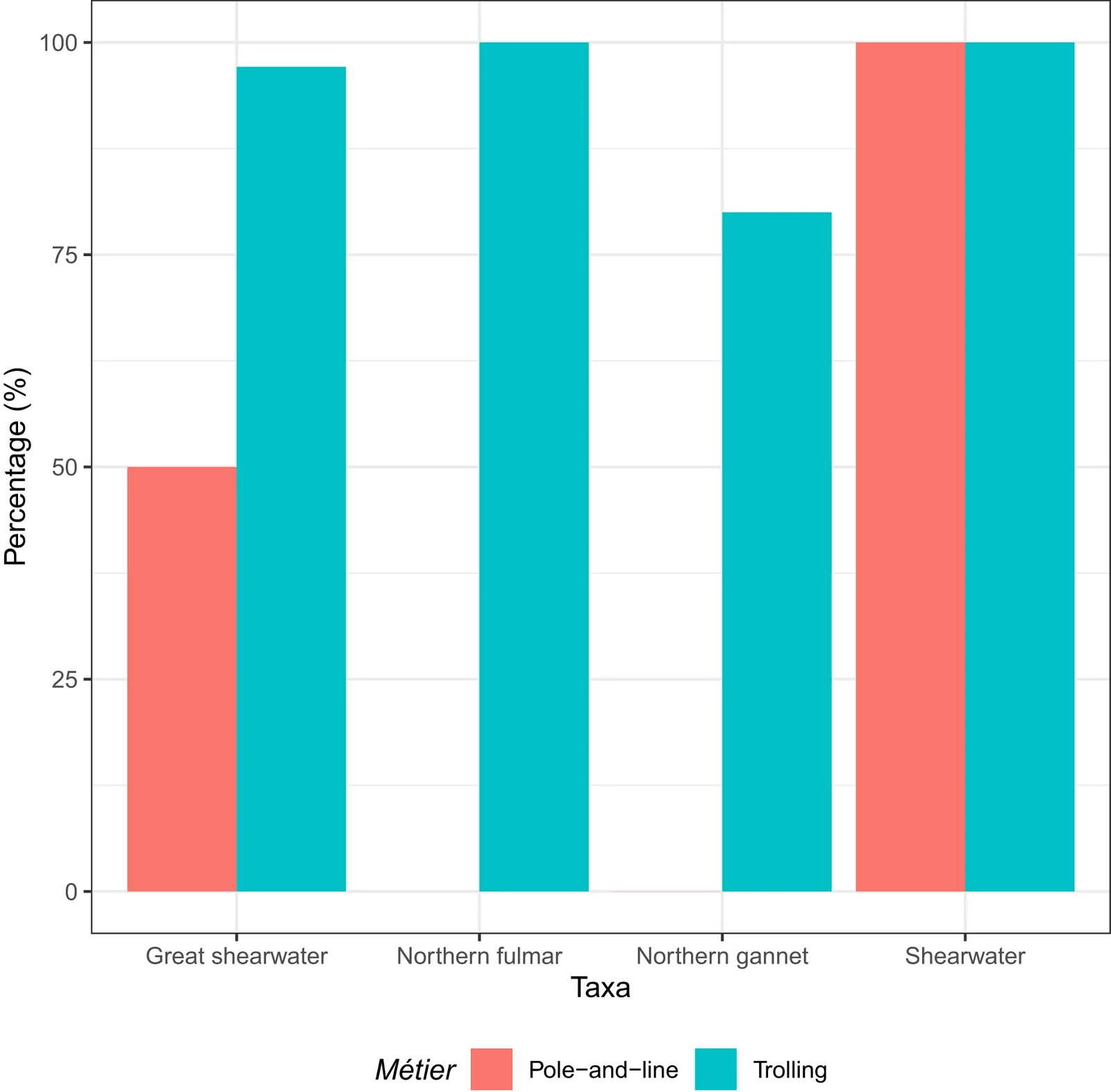
Impact of the bycatch of endangered, threatened and protected taxa in the artisanal tuna fishery of the Bay of Biscay (2016–2019). The barplot shows the percentage of individuals released alive by taxa and *métier*.

According to the conservation frameworks consulted (see ‘Methods’), all three bycaught species fall under several national, European and global conservation assessments. At the national level, they are included in Spain’s *List of Wild Species under a Special Protection Regime*. At the European level, they are protected under the EU Birds Directive, although none is listed under Annex I. All three species are also prioritised under Biodiversity Descriptor 1 of the MSFD, and none appears on the OSPAR List of Threatened and/or Declining Species. Under the regional IUCN European assessment, the northern gannet is classified as *Least Concern*, while the northern fulmar is considered *Threatened*; no regional assessment is available for the great shearwater. At the global level, the IUCN Red List classifies all three species as *Least Concern*.

## Discussion

This study provides the first systematic assessment of ETP species occurrence and bycatch in the artisanal tuna fishery operating in the Bay of Biscay (BoB) and adjacent waters. Although a wide diversity of megafauna was observed around fishing operations, only three seabird species were recorded as bycatch, and no captures of marine mammals occurred during monitored trips. These results reflect the operational characteristics of this small-scale fishery, which uses single-hook gear under close visual control. Nevertheless, occasional captures of marine mammals or turtles remain plausible, and their detection would require continued monitoring capable of capturing rare events and interannual variability.

The high selectivity of the fishery was further reflected in catch composition: trolling retained 99.9% target albacore, with only ~0.1% discards of other tuna species (bigeye and occasional bluefin), consistent with long-term observations from fleets in the BoB (de Zárate et al., 2005; de Zárate, Pérez & Quelle, 2015; Santiago et al., 2016a). Similarly, pole-and-line retained 100% tuna catch, targeting mostly albacore and bluefin. The only discards in pole-and-line operations arose during live bait purse-seine sets, where slipping practices released nonbait fish alive; this aligns with previous studies reporting high post-release survival of small pelagic fishes when slipping is performed under controlled conditions (Arregi et al., 2014; Scientific, Technical and Economic Committee for Fisheries (STECF), 2014). Taken together, these results consolidate the evidence that both *métiers* operate with exceptionally low discard levels compared to many European fisheries.

The diversity of megafauna species observed around the fleet should be interpreted within the broader ecological context of the BoB, one of the most megafauna-rich pelagic areas in the Northeast Atlantic. Recent ecosystem-wide assessments indicate that these habitats support diverse communities of cetaceans, shearwaters, gannets, and other pelagic predators occupying distinct trophic niches during summer (Louzao et al., 2019b; Iglesias et al., 2024). Likewise, largescale aerial surveys have shown that the BoB regularly hosts large whales, pilot whales, and substantial populations of common and striped dolphins concentrated along the steep shelf break, submarine canyons, and productive frontal systems characteristic of the region (Laran et al., 2017; Gilles et al., 2023). This ecological richness is consistent with the diversity of species observed around the artisanal tuna fishery. However, the limited number of species involved in bycatch events suggests that spatial co-occurrence does not necessarily imply bycatch with fishing gear under the operational conditions documented.

Within this ecological backdrop, differences between *métiers* become clearer. ETP bycatch events occurred more frequently in trolling than in pole-and-line operations under the monitoring coverage achieved, and the two *métiers* also differed in the number of birds involved per event. Pole-and-line events consistently involved one or, occasionally, a few individuals, whereas trolling produced a broader range of outcomes, including events in which several shearwaters were caught at once. The great shearwater was the principal species recorded as bycatch, with most individuals released alive. These captures occurred mainly in oceanic waters, aligning with the offshore distribution of the species and the spatial footprint of trolling, consistent with the susceptibility pattern suggested for great shearwaters by García-Barón et al. (2023). Unidentified shearwaters captured in similar offshore areas likely included great and Cory’s shearwaters (Astarloa et al., 2021), with possible contributions from sooty shearwaters (Louzao et al., 2019a). Northern gannets were recorded mainly in pole-and-line operations in shelf-slope waters during summer, matching their known seasonal distribution in the BoB (Pettex et al., 2017; Louzao et al., 2020). The single northern fulmar detected in trolling is consistent with its preference for shelf-slope habitats. Overall, these patterns reflect the distinct spatial and behavioural ecology of each species and the *métier*-specific fishing dynamics shaping both the frequency of events and the number of individuals involved per event.

The higher occurrence of bycatch in trolling can be explained by gear configuration and fishing behaviour. Continuously towed surface lures deployed at varying distances behind the vessel provide repeated opportunities for seabirds to approach and attempt to seize artificial baits, particularly under windy or turbulent conditions. When groups of shearwaters are present, such circumstances can occasionally result in multiple individuals being captured during the same event. In contrast, pole-and-line gear is handled directly alongside the vessel and is accompanied by continuous human activity, splashing and the release of live bait—factors that tend to deter birds from approaching hooks. Moreover, pole-and-line fishing is episodic and spatially confined, whereas trolling covers wider offshore areas over longer periods, increasing overlap with pelagic seabird aggregations. These mechanisms are consistent with observations from comparable fisheries: pole-and-line tuna fisheries elsewhere report minimal seabird bycatch because hooks are never left unattended at the surface (Restrepo et al., 2025), whereas surface-hook fisheries, including trolling, can occasionally result in events involving more than one bird when seabirds strike baited or artificial lures (Wiedenfeld et al., 2012; International Seafood Sustainability Foundation (ISSF), 2025).

Seasonal patterns of bycatch align closely with known species’ phenology in the region. The BoB is an important migratory area for ETP species, attracting long-distance migrants such as great shearwaters, which visit the region in late summer–autumn (August–October) mostly as nonbreeders (Hobbs et al., 2003; Louzao et al., 2015). Their arrival timing varies with climatic conditions, such as the North Atlantic Oscillation, which influences flight efficiency and feeding opportunities (Louzao et al., 2015). The timing and distribution of the great shearwater bycatch observed in this study—including events in which several individuals were captured during the same operation—match this phenology and coincide with the offshore footprint of trolling activity (García-Barón et al., 2023). Northern gannets also show predictable seasonal movements, breeding in northern Europe and migrating through or wintering in the BoB (Arcos et al., 2009; Fort et al., 2012; Pettex et al., 2017). Their tendency to form dense shelf aggregations in winter and more diffuse distributions in summer is consistent with the low-number events recorded in shelf-slope areas during pole-and-line operations. Overall, the seasonal overlap between species migrations and *métier*-specific fishing activity helps explain both the occurrence of bycatch events and the number of individuals involved in them.

Most bycaught birds were released alive, but post-release survival cannot be inferred solely from onboard observations. Previous studies indicate that delayed mortality may occur following capture, especially when birds experience exhaustion or injury. Tagging-based studies are needed to assess post-release survival and potential sublethal effects. In the meantime, consistent implementation of best-practice handling protocols—such as those described in the ACAP hook-removal guide (Agreement on the Conservation of Albatrosses and Petrels (ACAP), 2019)—remains essential. The MSC certification process for this fishery (Santiago et al., 2016a) established requirements related to handling and monitoring practices that are relevant for ETP bycatch mitigation, although ensuring their uniform uptake across vessels and throughout the season remains important.

Improving the representativeness of ETP bycatch estimates in this fishery will require sustained sampling that captures the operational diversity of both *métiers*. In this study, observer coverage was very low for trolling (1.7%) and only slightly higher for pole-and-line (3.3%), resulting in an overall coverage below 3%. Such levels are insufficient for deriving reliable fleet-wide estimates of ETP bycatch, as low and uneven observation effort can lead to wide confidence intervals and potential sampling bias, particularly when sampling is opportunistic rather than probabilistic (Gilman, Passfield & Nakamura, 2012). Although ICCAT (Rec. 16-14) establishes a 5% minimum observer coverage, several evaluations have shown that even this level may not support accurate estimation of rare incidental events. Simulation analyses and expert recommendations (Babcock, Pikitch & Hudson, 2003; Agreement on the Conservation of Albatrosses and Petrels (ACAP), 2019) consistently highlight that substantially higher coverage is necessary to produce robust and representative bycatch estimates. VMS and logbook data provide high-resolution fishing-effort information that can support raising procedures once representative coverage is achieved, but under the current sampling conditions any extrapolation would yield excessively uncertain values. For these reasons, we did not attempt to raise the observed bycatch to fleet-wide estimates.

Future estimation efforts would benefit from expanded onboard observer coverage or the integration of Electronic Monitoring (EM), which has demonstrated strong consistency with human-observer data in this fishery. Higher and more representative coverage, combined with the detailed operational effort, would enable the use of statistically robust raising procedures or model-based estimation approaches and thereby improve confidence in total bycatch estimates across both *métiers*. EM represents a particularly practical avenue for increasing effective observation coverage. All tuna RFMOs, including ICCAT, have recently established minimum EM standards, and EM trials onboard trolling and pole-and-line vessels in this fishery have shown excellent agreement with observer records (Villar Arribi, 2020; Ruiz et al., 2021). EM has been integrated into the monitoring programme since 2020, and further expansion in the coming years—combined with onboard observers and existing effort data—will substantially strengthen the ability to characterise ETP bycatch and support more comprehensive and representative monitoring strategies.

In conclusion, this study provides the first comprehensive assessment of ETP species occurrence and bycatch in the artisanal tuna fishery of the Bay of Biscay and adjacent waters. Observers documented notable megafauna richness around fishing operations, although bycatch events involved only three seabird species. The results reveal *métier*-specific patterns shaped by the spatial and operational characteristics of the fishery and the seasonal distributions of ETP species. Importantly, the study shows that evaluating bycatch requires considering both how often events occur and how many individuals may be involved in a given event, as trolling occasionally resulted in events with multiple individuals, whereas pole-and-line events consistently involved very few birds. Continued monitoring—through onboard observers and electronic monitoring—together with consistent implementation of best-practice handling protocols, will be essential for documenting ETP bycatch, minimising risk, and supporting adaptive management. These efforts are particularly relevant given the differing conservation statuses of the bycaught species and the need for robust baselines to evaluate future environmental and fisheries-related changes.

## Supplemental Information

10.7717/peerj.21516/supp-1 Supplemental Information 1 List of bycaught Protected, Endangered and Threatened (PET) species. Detailed information for each PET bycatch event, including métier, date, geographic position, common and scientific names of the species, and the number of individuals captured per fishing haul.

## Additional Information and Declarations

### Data Availability

The following information was supplied regarding data availability:

The data is available in the Supplemental File.
