# Supplementary material for: Assessing selectivity and bycatch of endangered, threatened and protected species in artisanal tuna fisheries in the NE Atlantic
Source: PeerJ. 2026 Oct 30;14:e21516. doi: 10.7717/peerj.21516 (PMC13726573; doi:10.7717/peerj.21516)
Supplement: Supplementary file 1 [file peerj-14-21516-s001.docx]

Metier	Date	Lat	Long	Common name	Scientific name	Nºind.
Pole and Line	22/09/2016	43°58´N	04°25´W	northern gannet	Morus bassanus	3
Pole and Line	02/09/2016	46°31´N	04°25´W	northern gannet	Morus bassanus	1
Pole and Line	02/08/2017	44°45´N	4°35´W	shearwater	NA	1
Pole and Line	06/08/2017	45°03´N	4°01´W	great shearwater	Ardenna gravis	2
Pole and Line	31/07/2018	44°45´N	4°35´W	great shearwater	Ardenna gravis	1
Pole and Line	31/07/2018	45°42´N	5°01´W	great shearwater	Ardenna gravis	1
Pole and Line	31/07/2018	45°45´N	4°54´W	great shearwater	Ardenna gravis	1
Trolling	13/08/2018	48°45´N	11°32´W	northern gannet	Morus bassanus	1
Trolling	18/08/2018	48°10´N	09°59´W	great shearwater	Ardenna gravis	1
Trolling	14/08/2018	48°18´N	10°17´W	great shearwater	Ardenna gravis	1
Trolling	15/08/2018	48° 34´N	10°55´W	great shearwater	Ardenna gravis	1
Trolling	16/08/2018	48°45´N	11°32´W	great shearwater	Ardenna gravis	5
Trolling	17/08/2018	48°45´N	11°32´W	great shearwater	Ardenna gravis	5
Trolling	05/07/2018	45°24´N	03°27´W	northern gannet	Morus bassanus	1
Trolling	05/08/2018	47°59´N	10°16´W	great shearwater	Ardenna gravis	1
Trolling	07/08/2018	48°45´N	10°18´W	great shearwater	Ardenna gravis	3
Trolling	07/08/2018	48°45´N	10°18´W	northern fulmar	Fulmarus glacialis	1
Trolling	17/08/2018	46°50´N	06°02´W	great shearwater	Ardenna gravis	1
Trolling	03/08/2018	45°59´N	05°06´W	great shearwater	Ardenna gravis	15
Trolling	06/08/2018	48°04´N	10°40´W	great shearwater	Ardenna gravis	1
Trolling	09/08/2018	48°20´N	10°01´W	great shearwater	Ardenna gravis	1
Trolling	19/06/2019	44°34´N	03°18´W	northern gannet	Morus bassanus	1
Trolling	19/06/2019	44°36´N	03°20´W	northern gannet	Morus bassanus	1
Trolling	27/08/2019	48°20´N	10°06´W	shearwater	NA	3
Trolling	27/08/2019	47°50´N	09°21´W	northern gannet	Morus bassanus	1
Trolling	27/08/2019	47°45´N	09°13´W	shearwater	NA	4
Trolling	27/08/2019	48°34´N	10°28´W	shearwater	NA	1
